# Shamed If You Do, Shamed If You Do Not: Group-Based Moral Emotions, Accountability, and Tolerance of Enemy Collateral Casualties

**DOI:** 10.3389/fpsyg.2022.750548

**Published:** 2022-03-02

**Authors:** Noa Schori-Eyal, Danit Sobol-Sarag, Eric Shuman, Eran Halperin

**Affiliations:** ^1^Baruch Ivcher School of Psychology, The Interdisciplinary Center (IDC) Herzliya, Herzliya, Israel; ^2^Psychology Department, Hebrew University of Jerusalem, Jerusalem, Israel; ^3^Faculty of Behavioural and Social Sciences, University of Groningen, Groningen, Netherlands

**Keywords:** accountability, group-based emotions, intergroup conflicts, civilian casualties, shame, guilt

## Abstract

Civilian casualties contribute to the perpetuation of intergroup conflicts through increased radicalization and hostilities, but little is known on the psychological processes that affect responses to outgroup civilian casualties. The goal of the present research was to explore two factors expected to lead group members to act more cautiously, thereby reducing civilian casualties: perceived accountability and forecast group-based moral emotions. In two studies, Jewish–Israeli civilians (Study 1) and soldiers (Study 2) were asked to forecast their group-based moral emotions in case of Palestinian (i.e., outgroup) civilian casualties, then exposed to accountability manipulations. Participants who expected to feel low levels of shame and were primed with accountability made more cautious decisions than those in the control condition. Participants who expected to feel high levels of shame were unaffected by accountability primes. Theoretical and practical implications regarding forecast moral emotions and accountability as an intervention in intergroup conflicts are discussed.

## Introduction

A little past midnight of Midsummer, 21 June 2016, Muhammad Rafat Badran, a 15-year-old Palestinian was traveling by car with his cousins in the Central West Bank. The boys were returning from an outing to a swimming pool in the village of Beit Sira, not far from Ramallah, when their car was mistakenly identified by the Israeli Defense Forces (IDF) to be involved in throwing rocks and firebombs. The soldiers shot multiple times at the suspect vehicle: Muhammad was killed immediately, and his cousins were wounded (Harel et al., 2016). This incident is but one example of how uninvolved civilian casualties can result from errors and misjudgments, with tragic results.

Civilian casualties often occur during severe intergroup conflicts, despite rules of conduct such of the International Humanitarian Law that are designed to minimize them. While it is difficult to gauge actual numbers of civilian casualties (e.g., Ryan, 2018; Crawford and Lutz, 2019), evidence has accumulated that collateral damage—unintentional or incidental injury or damage to persons or objects that would not be lawful military targets in the circumstances ruling at the time (U.S. Joint Chiefs of Staff, 2013)—can have a deleterious impact on intergroup conflicts beyond the obvious human suffering and financial burden. The incidental death of non-combatants can contribute to the perpetuation and even escalation of conflict through increased radicalization and more negative attitudes toward the rival outgroup (e.g., Condra and Shapiro, 2012; Deri, 2012; Lyall et al., 2013; Shaver and Shapiro, 2015; Farooq et al., 2020; though see Shah, 2018).

Existing research focused on public attitudes toward enemy civilian casualties, yielding conflict results: Some opinion polls and lab experiments revealed an aversion to civilian casualties (e.g., Friedrich and Dood, 2009; Kreps, 2014; Pew Research Center, 2015, 2017; Walsh, 2015; Johns and Davies, 2019), whereas others found a more lenient view on the issue (Larson and Savych, 2007; Gallup, 2011; Sagan and Valentino, 2017; Carpenter and Montgomery, 2020; Slovic et al., 2020). In the present research, we explore a different angle of ingroup members’ answer to the question of potential outgroup civilian casualties. We go beyond mere attitudes regarding collateral casualties or support for macro-level policies and focus on group members’ decisions in situations, in which *they* would be required to make an immediate, life-or-death response that might result in the incidental killing of uninvolved civilians. In situations of violent conflicts that involve terror attacks on civilians, such occurrences can become real all too often. We focus on two elements that may affect the decision-making process and reduce tolerance of collateral casualties: accountability and forecast group-based moral emotions, while exploring possible connection between them.

## Accountability and Tolerance of Civilian Casualties

Accountability refers to an implicit or explicit expectation that one may be called upon to justify one’s beliefs, feelings, and actions to others (Scott and Lyman, 1968; Semin and Manstead, 1983; Tetlock, 1992). Accountability can be conceptualized as a primarily social phenomenon in which individuals seek to maintain prestige and avoid “losing face” to any potential observers of their actions. It usually implies that individuals who do not provide an adequate justification for their actions will suffer negative consequences, ranging from scornful stares to loss of livelihood, liberty, or even life (Stenning, 1995). Conversely, people who provide satisfactory justifications will experience positive consequences, ranging from mitigation of punishment to lavish rewards, like a prestigious position in political office (Lerner and Tetlock, 1999). This conception of accountability expands its relevance to nearly every action or belief of an individual that could be witnessed or observed by others, especially on socially or politically relevant issues (Wayne et al., 2016).

The effect of accountability on decision-making has been tested in multiple fields, including medicine (Han et al., 2009), business negotiations (Lerner and Shonk, 2006), tax audits (Buchman et al., 1996), postwar aid (Skitka et al., 1991), education (e.g., Miller, 1995; Fuhrman and Elmore, 2004; Burke, 2005), the criminal justice system (Stenning, 1995), and representative government (e.g., Przeworski et al., 1999; Behn, 2001; Grant and Keohane, 2005). Accountability that meets certain criteria can promote thoughtful and careful consideration of the merits of a specific attitude or preference (Lerner and Tetlock, 1999). This form of accountability, which leads to “preemptive self-criticism” (Tetlock, 1983, p. 81), may therefore induce people to choose a more cautious course of action when confronted with potential aggressors, thus reducing the risk of civilian casualties. Thus, we expect that when individuals experience a sense of accountability, they would be less tolerant of enemy collateral casualties compared with situations, in which no perception of accountability is evoked.

## Group-Based Moral Emotions

The term “preemptive self-criticism” can also depict another factor that may impact ethical decision-making: forecast group-based moral emotions. Moral emotions influence the link between moral standards and moral behavior, driving people to behave in moral, socially appropriate ways in their social interactions and intimate relationships (Retzinger, 1987; Baumeister et al., 1994; Leith and Baumeister, 1998). Self-reflection evokes emotions that provide immediate reinforcement, either positive or negative, of behavior—not only behavior that already took place, but also planned behavior and its expected outcomes (Tangney et al., 2007). By providing immediate and salient feedback on our social and moral acceptability, moral emotions are a significant force in regulating behavior.

In the context of intergroup relations and intergroup conflict, shame and guilt are arguably some of the most relevant moral emotions. Group-based guilt is associated with appraised responsibility of one’s ingroup for moral violations (Branscombe, 2004); it is evoked when one feels personally or collectively complicit in other group members’ transgressions (Lickel et al., 2011), or actions that are seen as illegitimate (Branscombe et al., 2002) and can motivate group members to rectify the wrongdoing and make reparations to the victims (e.g., Doosje et al., 1998; Iyer et al., 2003; Čehajić et al., 2011). Group-based shame, on the other hand, is associated with appraisals implying that a wrongdoing tarnishes the moral image of the group (Lickel et al., 2011). Group-based shame was shown to induce a desire to distance the ingroup from the shame-invoking situation (Lickel et al., 2004; Iyer et al., 2007), yet there are inconsistent findings regarding its association with pro-social motivations (Rees et al., 2013; Berndsen and Gausel, 2015) and pro-reconciliation actions, such as expressing contrition over wrongdoings and compensating the outgroup (Brown and Čehajić, 2008; Allpress et al., 2010; Gausel et al., 2012).

The majority of research on group-based shame and guilt focuses on the emotions evoked either by past transgressions or present moral violations. However, self-conscious emotions can provide feedback regarding future actions, and whether those actions comply with one’s moral standards and values (Tangney et al., 2007). Forecast group-based moral emotions—that is, *expected* shame and guilt over future wrongdoings—can also impact on group members’ future actions. This notion received empirical support from a series of studies conducted by Shepherd et al. (2013a), in which expected group-based shame was associated with less ingroup favoritism, more egalitarian intergroup behavior (Shepherd et al., 2013b), and increased support for collective action and willingness to make reparations (Shepherd et al., 2013c). We therefore expect that forecasting higher levels of group-based guilt and shame over expected collateral casualties would be associated with lower support for actions that would lead to such casualties.

Furthermore, shame is driven by the damaged reputation or loss of respect and honor in the eyes of others (Crozier, 1998; Mosquera et al., 2002; Smith et al., 2002), which resonates with the concept of accountability. This suggests that forecast moral emotions and accountability may operate in tandem to impact the decision whether to take an action that may result in the death of outgroup civilians or to refrain from action at the possible cost of ingroup lives. Shame, in particular, can be construed as a form of internalized accountability system. We therefore expected that forecast group-based moral emotions would moderate the effect of the accountability manipulation. One possible outcome was additive: The most cautious decisions would be made by individuals who expected to feel high levels of guilt and shame over outgroup civilian casualties *and* were exposed to an accountability manipulation. Another potential outcome is derived from the notion that shame, in particular, can be construed as a form of internalized accountability system; therefore, it is possible that individuals who already forecast high levels of shame would be less affected by accountability manipulations. Both possible directions of the moderation were explored. Finally, we tested the possibility that forecast group-based guilt would also moderate the effect of the accountability manipulation, either making participants even more reluctant to put outgroup civilians at risk when primed with accountability, or making the manipulation redundant.

## Current Research

To test our hypotheses, we conducted two studies in the context of the Israeli–Palestinian conflict.^1^ In Study 1, which was conducted in the lab, Jewish–Israeli students were first asked to forecast their level of group-based emotions in case of incidental deaths of enemy civilians, then underwent a traditional accountability manipulation, and responded to a set of vignettes presenting potential attackers/bystanders. In Study 2, Israeli Defense Force (IDF) soldiers and officers were recruited to respond to a modified series of vignettes adapted to their role as likely first responders on the scene; accountability was primed by requesting participants to describe military debriefings they had undergone during their military service. We expected that high levels of forecast moral emotions would lead to more cautious responses that decrease the chance of incidental civilian casualties. We also expected that priming a sense of accountability would lead to more cautious decision-making compared with no intervention. Finally, we examined the possibility that the accountability manipulation would have a different effect on participants with lower levels of forecast moral emotions than on those with higher levels of forecast moral emotions, which represent internalized accountability.

## Study 1: Testing the Proposed Model

Study 1 was conducted at the height of the “Knife Intifada,” often referred to as “Lone Wolves Intifada” in the Israeli media, while Palestinian attacks against Jewish–Israeli civilians and security forces were almost daily occurrences. This period of conflict escalation began in September, 2015 and lasted several months, in which dozens of Palestinian attacks were attempted against Jewish–Israeli soldiers and civilians. According to Israeli reports, between October, 2015 and October 2016 there was a total of 166 stabbing attacks and 89 attempted stabbings; 108 shootings; 47 vehicular (ramming) attacks; and one vehicle bus bombing (Israel Ministry of Foreign Affairs, 2018). The attacks occurred in numerous towns and settlements in Israel and were directed at both civilians and soldiers, thus exposing much of the population to the threat of attack. Most Palestinian “lone wolves” were young (their median age was 20) and who did not seem to be backed by a broad Palestinian consensus, nor were the Palestinian political organizations much involved in the uprising (Chorev, 2019). During this period, nearly 50% of Palestinian attackers were killed; in some incidents, Israeli soldiers and members of other security forces allegedly used excessive force to “neutralize” attackers or suspect attackers (B’Tselem, 2015). This combination of circumstances made salient both the sense of imminent threat and the potential mistaken response that could cost civilian lives.

Against this backdrop we conducted Study 1. The goal of the study was to examine the effects of accountability and forecast moral emotions on the decision whether or not to choose an action that may lead to enemy civilian casualties. This decision was operationalized by presenting Jewish–Israeli participants with ambiguous vignettes describing possible terror attacks and requesting them to decide whether the potential attacker in each vignette should be shot.

### Participants and Procedure

Seventy-three Jewish–Israeli students participated in the study in exchange for course credit.^2^ The sample included 11 men and 62 women whose ages ranged between 20 and 44, *M* = 22.89, *SD* = 2.90. The study was conducted in three sessions. In the first online session, which was part of a larger survey, participants completed a demographics section that included a measure of political orientation. Political orientation was included as control measure because of its connection with support for aggressive policies in intergroup conflict in general (e.g., Sibley and Duckitt, 2008; Jost et al., 2009) and with tolerance of enemy civilian casualties in particular (e.g., Uhlmann et al., 2009; Schori-Eyal et al., 2019). In the second online session, approximately 3 weeks later, participants forecast their group-based emotions regarding a scenario, in which uninvolved civilians are inadvertently harmed during an attempt to stop a terror attack. In the third and final session, conducted in the lab 24–48 h later, participants were randomly assigned to either the accountability condition or the control group (anonymity). After a short explanation, which served either as a manipulation or control, participants were presented with eight vignettes describing possible terrorist attacks similar to the description they had read in the first session and were asked to make a decision about each vignette. Finally, participants were debriefed by the experimenter.

### Measures

At T1 political orientation was measured using the item “how would you describe your political orientation?” ranging from 1 (radical left) to 7 (radical right). At T2 forecast group-based emotions were measured by requesting participants to assess the degree to which they expect to feel different emotions if civilians were incidentally harmed during a terror attack (see Supplementary Material for all full measures). Participants then responded on a scale of 1 (not at all) to 6 (very much) regarding 11 emotions, including shame and guilt over harming uninvolved civilians.^3^

At T3, we carried out an accountability manipulation based on Lerner et al. (1998) and its adaptation by Wayne et al. (2016). Participants assigned to the accountability condition were told by the experimenter that after completing the questionnaire they would be interviewed by another researcher, who would ask them to elaborate on the rationale behind their decisions. Participants in the control condition were told that their decisions and answers will remain completely anonymous.

Next, we measured tolerance of enemy collateral casualties (TECC) using eight short vignettes. The vignettes were based on real events that occurred during the “Knife Intifada” (2015) and depicted mostly ambiguous situations that could be construed as a terror attack or of an obvious attack with a possible (but not certain) attacker. The number of indicators that the target was indeed an attacker was varied across the scenarios. After each vignette, participants were asked to determine whether the person described should be shot with the intent to kill. The responses were yes/no.

Based on participants’ responses to the vignettes we calculated a bias measure (c), based on the standard signal detection formula (Stanislaw and Todorov, 1999), which averages the z-score corresponding to the hit rate and the false alarm rate, with a loglinear correction for extreme values (Hautus, 1995; Stanislaw and Todorov, 1999; see Supplementary Material for detailed explanation). Positive responses were coded as false alarms for all scenarios (i.e., they were treated as noise trials), except for the most extreme scenario, where there was considerable evidence that the target was indeed the attacker. For this scenario, a positive response was coded as a hit (i.e., this was treated as a signal trial).^4^ Positive scores indicate a cautious bias, with higher scores indicating a more cautious bias, a score of 0 indicates a neutral bias, whereas negative scores indicate a lax bias, with lower scores being even more lax.

### Results and Discussion

Means, SDs, and zero-order correlations of variables in Studies 1 and 2 are presented in Table 1. To test our predictions of the effects of accountability, forecast group-based moral emotions, and a possible interaction between them, PROCESS Model 1 of Hayes (2013) was used to predict TECC, measured by the bias in the decision whether the target should be shot with intent to kill. Guilt and shame were tested as possible moderators in separate analyses. We controlled for political orientation in both studies.^5^

**Table 1:** Means, standard deviations and zero-order correlations between variables (studies 1-2).

S. no			Mean (*SD*)	1	2
1.	Forecast guilt	Study 1	4.11 (1.57)		
		Study 2	2.23 (1.23)		
2.	Forecast shame	Study 1	3.79 (1.58)	0.51^**^	
		Study 2	2.32 (1.28)	0.73^**^	
3.	Tolerance of enemy collateral casualties	Study 1	1.45 (0.19)	0.11	0.27^*^
		Study 2	1.17 (0.35)	−0.01	0.1
4.	Political orientation	Study 1	3.93 (1.34)	0.1	0.26^*^
		Study 2	3.48 (1.24)	0.37^**^	0.40^**^

* p < 0.05 and

** p < 0.01.

**Figure 1:**
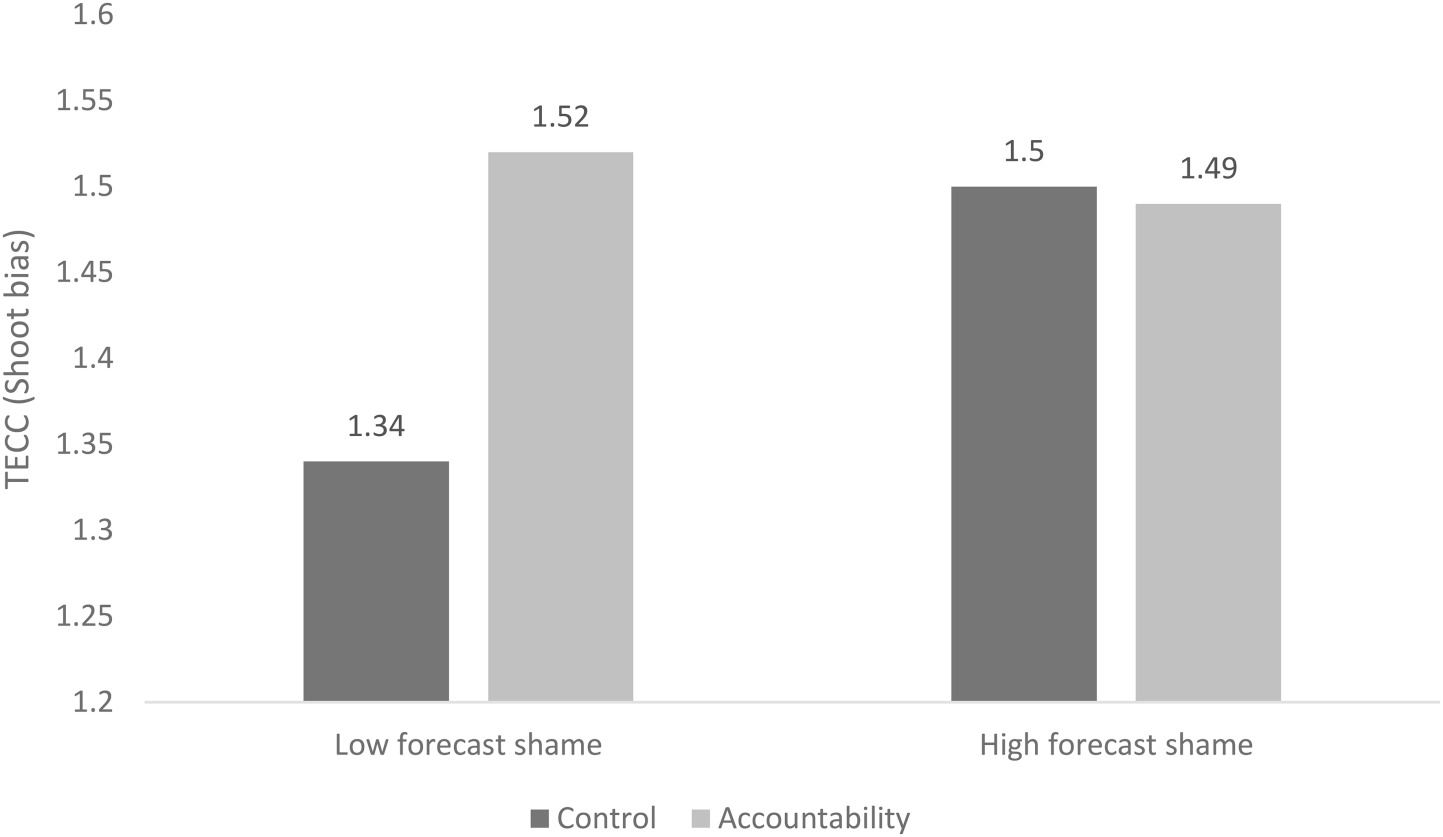
Interaction between forecast group-based shame and accountability predict tolerance of enemy collateral casualties as decision-making bias in Study 1.

Results of the analysis with forecast shame as a moderator [*r*^2^ = 0.24, *F* (4, 66) = 5.28, *p* < 0.001, change in *r*^2^ = 0.05] when the interaction term was added to the model indicated a marginally significant main effect of the experimental condition: participants in the accountability condition tended to be less likely to choose the “shoot” option compared with those in the control condition (*b* = 0.07, *SE* = 0.04, *t* = 1.89, *p* = 0.06). Forecast shame was not a significant predictor (*b* = 0.01, *SE* = 0.02, *t* = 1.14, *p* = 0.26). Taking into account the main effects, the interaction between forecast shame and experimental condition was a significant predictor of TECC (*b* = −0.05, *SE* = 0.02, *t* = −2.07, *p* = 0.04).^6^ Analysis of the simple effects (see Figure 1) indicated that while the experimental condition did not affect participants with high levels of forecast shame (*b* = −0.001, *SE* = 0.06, *t* = 0.20, *p* = 0.84), participants with low levels of forecast shame who expected to be held accountable were less likely to make the “shoot” decision compared with those in the control condition (*b* = 0.16, *SE* = 0.06, *t* = 2.81, *p* < 0.01). The difference between low- and high-forecast shame was significant in the control condition (*b* = 0.04, *SE* = 0.02, *t* = 2.39, *p* = 0.02), but not significant in the accountability condition (*b* = −0.01, *SE* = 0.02, *t* = −0.51, *p* = 0.61). When we conducted the analysis with guilt as a moderator [*r^2^* = 0.18, *F* (4, 66) = 3.67, *p* = 0.01], only accountability was a significant predictor of TECC (*b* = 0.08, *SE* = 0.04, *t* = 2.05, *p* = 0.045). Forecast group-based guilt did not have a main effect (*b* = 0.003, *SE* = 0.01, *t* = 0.23, *p* = 0.82) and did not moderate the effect of accountability on TECC (*b* = −0.02, *SE* = 0.03, *t* = −0.81, *p* = 0.42).

We decided to conduct an additional analysis, in which the TECC measure was composed of the sum of “shoot” decisions. The goal of this analysis was to examine whether the pattern of results remained similar when tolerance of enemy civilian casualties is operationalized as the total number of times in which participants chose to shoot a suspect. We found that participants in the accountability condition made fewer “shoot” choices in total compared with those in the control condition (*b* = −1.09, *SE* = 0.42, *t* = −2.59, *p* = 0.01). Forecast shame was also a significant predictor (*b* = −0.17, *E* = 0.1, *t* = 2.14, *p* = 0.04). Taking into account the main effects, the interaction between forecast shame and experimental condition was a significant predictor of TECC (*b* = −0.22, *SE* = 0.1, *t* = 2.14, *p* = 0.04). The difference between low- and high-forecast shame was significant in the control condition (*b* = −0.66, *SE* = 0.25, *t* = −2.65, *p* = 0.01), but not significant in the accountability condition (*b* = 0.12, *SE* = 0.24, *t* = 0.48, *p* = 0.63). The total model was significant [*r*^2^ = 0.48, *F* (4, 66) = 4.84, *p* < 0.01]. When we conducted the analysis with forecast guilt as a moderator, it did not predict this measure of TECC nor moderate the effect of the manipulation.

The results are in line with existing literature on accountability and its impact on decision-making. Priming accountability led participants to make more cautious decisions and fewer “shoot” choices in the ambiguous situations presented. We also found that forecast group-based shame (but not guilt) over incidentally killing enemy civilians moderated the effect of accountability on tolerance of enemy collateral casualties, measured as the level of bias in the “shoot” decisions. Of the two potential directions of moderation, these results support the second one we considered, but not the additivity hypothesis. The pattern of results suggests that in this context, accountability, and forecast group-based shame have a similar effect on decision-making. Accountability is a social phenomenon (Tetlock, 1992), driven by the concern that one may “lose face” if unable to justify one’s choices to an observer. Similarly, one facet of shame is the concern that one’s actions (or the actions of one’s group) would tarnish the social image (Lickel et al., 2011). Group members who are already concerned with the impact of the unintentional killing of uninvolved civilians on the group’s reputation and image—that is, those who expect to feel shame—are unaffected by the accountability manipulation.

One possible explanation is that the lack of effect on high-shame could be the result of a ceiling effect, as all participants were quite conservative in their decision-making and often chose not to shoot. Alternatively, these results could imply that for individuals who expect to feel shame, accountability is superfluous; they already take the possible negative consequences, namely the collective loss of face over incidental killing of uninvolved civilians, into account, regardless of whether or not they are held accountable for their decisions by an external judge.

How, though, can we explain the finding that expecting to feel guilt did not yield a similar effect? Even in the tense, hostile atmosphere of violent conflict escalation, the inadvertent killing of uninvolved enemy civilians was expected by our participants to induce similar levels of shame and guilt. It is possible, then, that forecast shame affects decisions in this context, but guilt remains separate from choosing a course of action. Mistakenly identifying a terrorist and killing bystanders may arouse group-based guilt, but not enough to alter decisions. In contrast, being concerned with the social cost of tarnished image and the negative judgment of others (either by expecting shame or feeling accountable) leads group members to choose more cautious actions.

Study 1 was carried out in a relatively small sample of college students, who were also characterized by a majority of women and a mostly centrist political orientation. In an attempt to replicate the results in a different sample and to test a different form of accountability, we conducted Study 2 among participants drawn from a unique population: soldiers and officers in active duty in the IDF, for whom the possibility of being in a position to stop a terror attack is highly feasible.

## Study 2: Priming Accountability Among Active Duty Soldiers

Based on the results of Study 1, we developed more nuanced hypotheses regarding the influence of accountability and forecast group-based emotions on tolerance of enemy collateral casualties, while taking into account the difference between the two moral emotions. Specifically, we expected that shame, but not guilt, would moderate the effect of the accountability manipulation on our outcome measures, so that those with lower levels of forecast shame would make more cautious decisions (i.e., fewer “shoot” choices) compared with those in the control group. We tested these hypotheses among active duty soldiers, who are more likely than the civilian sample of Study 1 to encounter the dilemma of possible collateral casualties, while trying to stop a terror attack. This was particularly true for our respondents as many of the, 2015–2016 terror attacks in Israel involved IDF soldiers—as targets, as those on scene and trained to stop an attack, or both. In Study 2, we evoked a sense of accountability by asking respondents to recall military debriefings or post-action reflective analyses.

The process of debriefing is an important part of military learning after missions. In “after-action review” practiced in the US Army (2011) participants talk through what happened in a military incident. In the IDF, soldiers and officers sit down in groups after battle to reconstruct what happened, a process in which the soldiers’ thoughts and feelings are considered part of the reality of combat (Shalev et al., 1998). When applied to—and tested in—a civilian context (surgical teams in a large hospital), debriefings were found to foster accountability norms (Vashdi et al., 2007). We therefore assumed that reminding participants who are members of the IDF of situations, in which they were debriefed would put them in a mindset of explaining and justifying their actions—in other words, make them feel more accountable.

### Method

#### Participants and Procedure

One hundred and fifty-seven IDF soldiers and officers volunteered to take part in the study.^7^^,^^8^ Thirty participants were recruited *via* social networks and completed the study online. The rest of the participants were approached on the train on their way to their army bases and completed a pen-and-paper questionnaire. The sample included 101 men and 46 women (10 participants declined to answer), whose ages ranged between 18 and 31, *M* = 20.00, *SD* = 2.50. Twelve respondents were career-track soldiers and officers; the rest were regulars on mandatory active duty. One hundred and twelve served either in combat or combat-support units; 27 respondents served in administrative units, and 25 chose not to answer the question. The mean duration of their military service was 16.08 months (*SD* = 10.66).^9^ Five participants identified as non-Jewish and were excluded from further analysis.^10^ No other participants were excluded.

Participants received the link to the questionnaire (if online) or were approached by a research assistant (if on the train). After completing the forecast emotions measures, participants were randomly assigned either to the accountability condition or the control condition. They wrote a short passage about military debriefings (accountability) or physical exercise in the military (control). Then, they read modified versions of the eight scenarios and made the decision whether the suspect should be shot. Then, they completed a short demographics questionnaire and were debriefed about the study.

### Measures

Forecast group-based emotions were measured using a modified version of the tool used in previous studies, focusing on the respondents’ potential role in a future military incident that could arouse these emotions. Participants were then presented with the list of eight emotions used in Study 2, including shame and guilt.^11^

The accountability manipulation was also modified to the context of military service. Participants in the accountability condition were asked to recall and write about two recent situations in which they were debriefed during their military service. Participants in the control condition were asked to recall two recent situations in which they exercised during their military service.

Tolerance of enemy collateral casualties. The eight vignettes were adapted to the military context with the aid of an IDF major from a combat unit. After each vignette, participants were asked “should you, as a soldier on the scene, shoot the person described with intent to kill?” and the bias measure was calculated accordingly. As in Study 1, we also tested our hypotheses with TECC measured as the sum of “shoot” choices.

### Results

**Figure 2:**
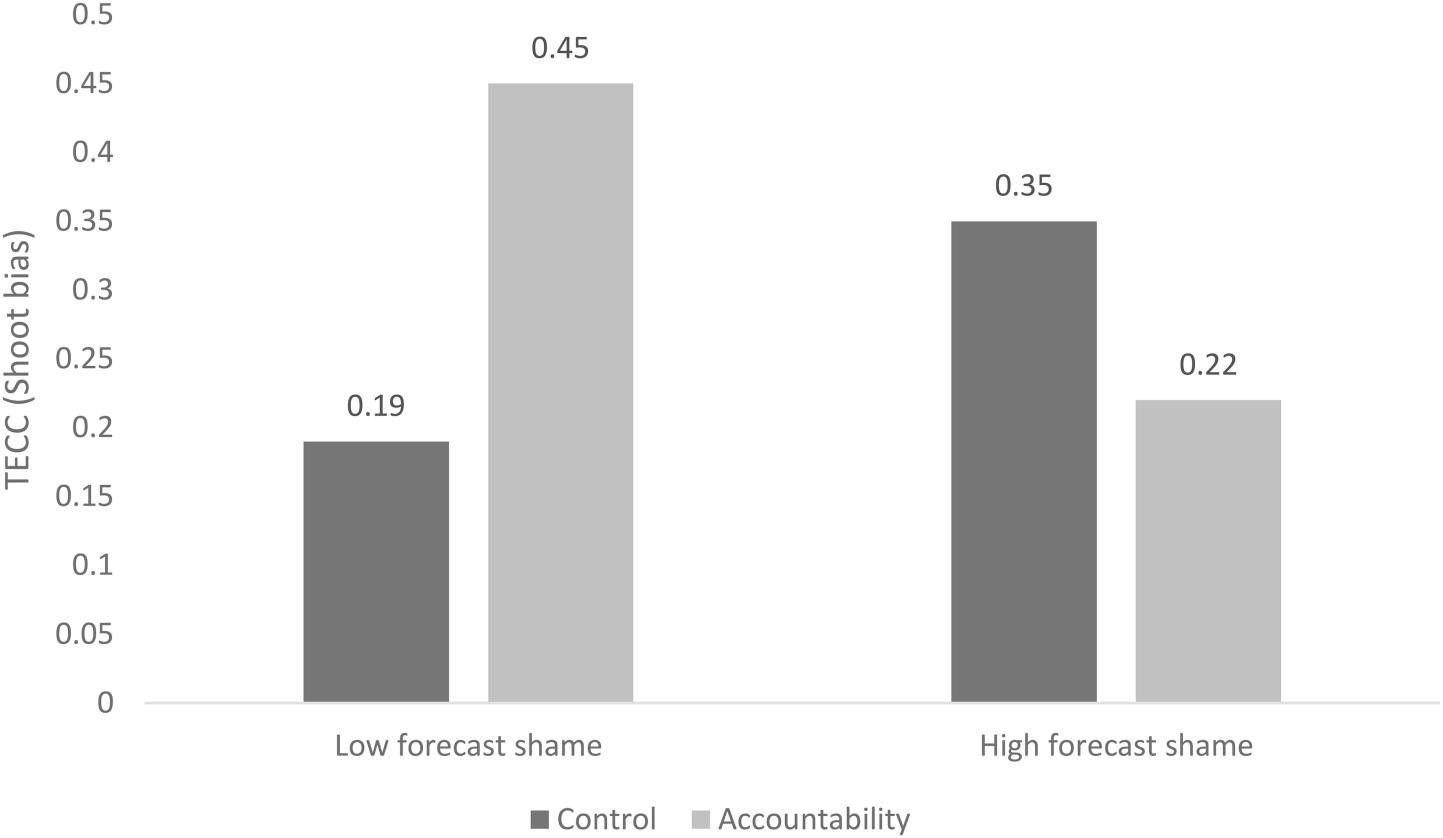
Interaction between forecast group-based shame and accountability predict tolerance of enemy collateral casualties as decision-making bias in Study 2.

As in Study 1, we used PROCESS Model 1 to test separately for forecast shame and guilt as moderators. The full model was significant [*r*^2^ = 0.11, *F* (4, 125) = 3.99, *p* = 0.005, change in *r*^2^ = 0.02 when the interaction term was added to the model]. In the first analysis and after taking into account the main effects of forecast shame and experimental condition, we found again that shame marginally moderated the effect of the accountability manipulation on the “shoot” bias (*b* = −0.13, *SE* = 0.07, *t* = −1.83, *p* = 0.07). Analysis of the simple effects (see Figure 2) revealed that participants with high levels of forecast shame were unaffected by the experimental condition (*b* = −0.13, *SE* = 0.16, *t* = −0.77, *p* = 0.44). Participants with low levels of forecast shame who had written about debriefings they had undergone during their military service tended to make fewer “shoot” decision compared with those in the control condition (*b* = 0.26, *SE* = 0.15, *t* = 1.81, *p* = 0.07). The difference between low- and high-forecast shame was not significant in the control condition (*b* = 0.05, *SE* = 0.05, *t* = 1.04, *p* = 0.30) or in the accountability condition (*b* = −0.07, *SE* = 0.05, *t* = −1.38, *p* = 0.17). Forecast shame (*b* = −0.01, *SE* = 0.04, *t* = −0.18, *p* = 0.86) and experimental condition (*b* = 0.09, *SE* = 0.11, *t* = 0.83, *p* = 0.41) were not significant predictors. When the analysis was conducted with TECC as the number of “shoot” decisions, we found a slightly different pattern of results: the accountability manipulation was a marginally significant predictor of this measure of TECC (*b* = −1.57, *SE* = 0.81, *t* = −1.94, *p* = 0.054), and forecast shame was not a significant predictor (*b* = −0.21, *SE* = 0.16, *t* = −1.31, *p* = 0.19). Forecast shame marginally moderated the effect of the accountability manipulation on the total number of “shoot” decisions (*b* = 0.4, *SE* = 0.22, *t* = 1.82, *p* = 0.07). The effect of the accountability manipulation was marginally significant among participants who forecast low levels of group-based shame (*b* = −0.77, *SE* = 0.45, *t* = −1.69, *p* = 0.09), and non-significant among those who expected to feel high levels of shame (*b* = 0.44, *SE* = 0.52, *t* = 0.85, *p* = 0.4). The entire model was significant [*r*^2^ = 0.13, *F* (4, 126) = 4.88, *p* = 0.001].

We conducted the same analysis with forecast guilt as the moderator. Neither forecast guilt (*b* = 0.02, *SE* = 0.04, *t* = 0.47, *p* = 0.64) nor the experimental condition (*b* = 0.08, *SE* = 0.11, *t* = 0.72, *p* = 0.47) nor the interaction between them (*b* = 0.04, *SE* = 0.07, *t* = 0.06, *p* = 0.96) were significant predictors of the shoot bias. A similar pattern was found with TECC as the sum of “shoot” decisions.

Study 2 replicated the effect of accountability priming among soldiers who expected to feel low levels of shame, inducing more cautious decisions in situations to which they, more than civilians, are exposed. Though, interestingly, the overall pattern of results differed from the previous study. Respondents who expected to feel high levels of shame tended to make *more* “shoot” choices when primed with accountability, and the experimental condition did not have the predictive effect found in the previous study. When TECC was assessed as the sum of “shoot” decisions, the results were more similar to Study 1 but the simple effect among low-shame participants was only marginal.

One possible explanation is that the accountability manipulation was not as strong a manipulation as the one used in Study 1; our respondents recalled situations in which they were debriefed, but did not expect to be held accountable (in contrast to Study 1 participants). The pattern of results may also have been affected by the social climate and the contrary messages to which Israelis, and particularly members of the security forces, were exposed at the time.

While the rules of engagement guiding IDF and other security forces had not been formally changed during the “Knife Intifada,” public discourse was leaning toward more use of lethal force. Calls to kill attackers in every situation were made by senior Israeli officials, such as MP Yair Lapid, head of a centrist party, who said: “Do not hesitate. Even at the start of an attack, shooting to kill is correct. If someone is brandishing a knife, shoot him.” Then, Minister of Public Security Gilad Erdan stated that “every attacker who sets out to inflict harm should know that he will likely not survive the attack”; and the commander of the Jerusalem Police Department at that time, Major General Moshe Edri, announced, “Anyone who stabs Jews or hurts innocent people is due to be killed.” In contrast, Chief of the General Staff Gadi Eisenkot, who was the Commander-in-Chief of the IDF during the Knife Intifada, gave a clear admonition to follow the Israeli military’s rules of engagement: “A soldier can only unlock the safety catch if there is a threat to him or his fellow soldiers. I do not want a soldier to empty a magazine on a girl holding scissors.”

Israeli Defense Force soldiers and officers have therefore been required to make split-second decisions on how to act while balancing conflicting messages. It is possible that asking soldiers to recall situations of debriefings led them to interpret their instinctive decisions as the “wrong” choice, over which they would be debriefed and possibly sanctioned. Thus, low-shame soldiers made more cautious decisions after being primed with accountability, whereas high-shame soldiers “corrected” more in the direction of shooting the suspects described in the vignettes. It is important to bear in mind, though, that the only significant simple effect found in Study 2 was consistent with the previous study, while other trends remained non-significant.

## General Discussion

In two studies conducted among Jewish–Israeli civilians and IDF soldiers, we examined the effects of accountability, forecast group-based moral emotions, and the interaction between them on tolerance of enemy collateral casualties. The results, we obtained suggest an interaction between forecast shame and accountability on tolerance of enemy civilian casualties. When primed with accountability, group members who did not expect to feel much shame over the incidental killing of uninvolved enemy civilians were more likely to choose not to “shoot” suspects in ambiguous situations. In contrast, group members who forecast high levels of shame were unaffected by experimental manipulations of accountability. Group-based guilt did not predict TECC, nor did its interaction with the accountability prime.

The results of the studies provide some support for our hypotheses, but it is important to acknowledge their limitations. The samples are relatively small, and although the pattern of interaction between accountability and forecast group-based shame was similar in both studies, the interaction was found significant in Study 1 and only marginally significant in Study 2. Thus, while these results are suggestive of an interesting direction, future research in larger samples is needed to replicate and confirm them. Despite these limitations, we offer some thoughts on what our findings suggest.

The findings of the two studies presented are in line with the notion that in some situations shame can be an adaptive emotion. Often construed as a highly aversive emotion that entails an appraisal of the self as fundamentally flawed (Lewis, 1971), much of the previous literature associated shame with a tendency to avoid failure and its consequences for oneself and others (Gilbert and Andrews, 1998; Tangney and Dearing, 2002; Tracy and Robins, 2004). However, more recent evidence shows that shame can also be linked to constructive approach orientation (e.g., Gausel et al., 2018; Mashuri and van Leeuwen, 2020), particularly in situations in which one’s failure, or one’s social image, seem more reparable (Leach and Cidam, 2015). When the failure or social image appear difficult to repair, suggest Leach and Cidam (2015, p. 984) in their meta-analysis, “What better response can there be to shame […] than to lessen one’s approach of it?” Killing uninvolved civilians is an irreparable act; thus, forecast shame over such detrimental future action can lead to avoiding the undesirable outcomes altogether by choosing the more cautious, and less deadly, option.

Yet, we found that forecast shame tended to have a moderating effect on tolerance of enemy collateral casualties rather than a main effect. As suggested earlier, this consistent relationship indicates that individuals who expect to feel high levels of shame are unaffected by the accountability manipulation, possibly because they have already internalized the external agent or judge to whom they need to justify their actions. Therefore, they tend to choose the more cautious option, regardless of whether they are primed to think of accountability or not. For those who forecast little shame over their group’s actions, accountability mimics the effect of shame by inducing them to make more choices that would not result in the outcome that would taint their group’s social image.

The interpretation of shame as internalized accountability also explains, at least in part, why accountability did not have a main effect on participants’ decisions in Study 2. For respondents who already experienced “preemptive self-criticism” (Tetlock, 1983) in the form of forecast shame, the priming of accountability was redundant and did not impact their decisions. Another possible explanation is that the subtle priming manipulation of Study 2 was not sufficient to produce a main effect, though it resulted in a conditional effect. This also suggests that accountability has the potential to be a useful tool to induce thoughtful and conscientious decision-making process in challenging and complex situations within intergroup conflict. Future research that further examines the intersection between moral emotions and accountability in this context, both among decision-makers and members of the public, could shed additional light on the underlying mechanisms and assist in developing accountability-based interventions.

While the moderated effect of accountability and its potential benefits are rooted in previous findings, the finding that forecast guilt did not affect tolerance of enemy collateral casualties is more puzzling. Why did guilt, long considered to be the more adaptive of the two moral emotions (e.g., Dearing et al., 2005; Tangney et al., 2007; Stuewig et al., 2010), have no effect on group members’ choice of action? Our findings are consistent with research that demonstrated that shame, but not guilt, affected outcomes such as support for collective action (Shepherd et al., 2013c), reduced ingroup favoritism (Shepherd et al., 2013a), and reconciliation orientation in violent reciprocal conflicts (Gausel et al., 2018). Moreover, it is possible that in the context of the decisions our participants were presented with, the expectation of guilt was mostly detached from the actual action taken. In other words, group members care about their ingroup’s image in this context (i.e., their expectation of shame) rather than about the actual moral aspects of inadvertently killing uninvolved enemy civilians (i.e., forecast guilt).

Finally, let us consider the manner in which tolerance of enemy collateral casualties was assessed. This concept is more traditionally assessed by directly asking participants to indicate the number of uninvolved civilians they would deem acceptable “collateral casualties” (e.g., Sagan and Valentino, 2017; Schori-Eyal et al., 2019), a measure that may be susceptible to social desirability (Krumpal, 2013; though see Carpenter and Montgomery, 2020). Our approach tackled the issue simultaneously more directly and more circuitously, by asking respondents about concrete, less hypothetical scenarios which are closer to their own reality. The tool we devised was uniquely tailored to the situation in which ingroup civilians—not necessarily members of the armed forces—found themselves likely to address such decisions. We contend, however, that the merits of applying SDT-based measures of TECC goes beyond the specific situation and can be usefully adapted to other contexts.

The present research provides some evidence on forecast moral emotions, accountability, and their potential impact on decision-making in the context of enemy collateral casualties. Future research that replicates these findings, examines the underlying mechanisms in greater detail and expands the scope of the research to additional contexts, while addressing the limitations of the current set of studies, would support our suggestion that these psychological processes can act in tandem to induce more moral decision-making in intergroup conflicts.

## Data Availability Statement

The raw data supporting the conclusions of this article will be made available by the authors, without undue reservation.

## Ethics Statement

The studies involving human participants were reviewed and approved by IRB, Interdisciplinary Center Herzliya. The patients/participants provided their written informed consent to participate in this study.

## Author Contributions

NS-E designed the research, collected, analyzed, and interpreted the data, and drafted and revised the manuscript. DS-S designed the research, collected and interpreted the data, and revised and approved the manuscript. ES took part in designing the research and the research tools, analyzed and interpreted the data, and revised and approved the manuscript. EH supervised the designing of the research, interpreted the data, and revised and approved the manuscript. All authors contributed to the article and approved the submitted version.

## Funding

This work was supported by the European Research Council (grant number 335607) to EH and the Israel Science Foundation (grant number 1585-2016) to NS-E.

## Conflict of Interest

The authors declare that the research was conducted in the absence of any commercial or financial relationships that could be construed as a potential conflict of interest.

## Publisher’s Note

All claims expressed in this article are solely those of the authors and do not necessarily represent those of their affiliated organizations, or those of the publisher, the editors and the reviewers. Any product that may be evaluated in this article, or claim that may be made by its manufacturer, is not guaranteed or endorsed by the publisher.

## Footnotes

^1^We also conducted a third study, in which accountability was manipulated by presenting participants with a text describing its benefits in organizational settings. This manipulation failed, and the study is included in the Supplementary Material.

^2^Since our main hypothesis was regarding moderation of the effect of accountability on forecast shame, we report sensitivity to detect an interaction in terms of change in the r-squared when the interaction term is added to the model. Assuming a two-tailed alpha of 0.05 and standard power (80%), we had power to detect on effect of change in r-squared of 0.09.

^3^The other emotions, we measured were anger at the Palestinians, pride, hatred, pity, happiness, empathy, fear, despair, at anger over harming uninvolved civilians. None of these emotions significantly predicted tolerance of enemy collateral casualties or moderated the effect of accountability on TECC.

^4^While our mean distribution of scenarios was uneven (i.e., it contained more false alarms than hits), this reflects our focus on when situations collateral causalities. In addition, changing this coding method does not affect the results as it simply shifts the entire distribution of bias scores.

^5^All variables were examined for outliers; two outliers (SD > 2.5) were found on the TECC measure (more than 2.5 mediations below the mean) and excluded from further analysis.

^6^One hundred and ninety-one soldiers and officers agreed to take part in the survey; those who did not complete it properly (i.e., did not write anything in response to the prompt or, among those who were recruited online, took over 30 min to complete the study) were excluded from analyses.

^7^Assuming a two-tailed alpha of 0.05 and standard power (80%), we had power to detect on effect of change in r-squared of 0.048.

^8^A relatively large number of respondents declined to provide personal information; we assume that this is because soldiers are encouraged to maintain secrecy about their military service.

^9^We assumed that the multiple identities and roles of non-Jewish soldiers, and the complexity of their position as members of the IDF in a conflict that primarily targeted Jews, would introduce additional variables that cannot be taken into account with a sub-sample of *N* = 5.

^10^Group-based pride, anger, fear, empathy, hatred, and remorse were also measured; none of them significantly moderated the effect of accountability on TECC. Hatred and empathy each predicted the level of TECC, but these findings are outside the scope of the present paper.

^11^In addition, the signal scenario, where shooting with the intent to kill was coded as the correct decision (i.e., a hit) was strengthened by adding a sentence that described the attacker was turning toward another person with the knife raised. This indicates an immediate danger to someone else which could justify the use of lethal force.

## References

[ref1] Allpress J. A. Barlow F. K. Brown R. Louis W. R. (2010). Atoning for colonial injustices: group-based shame and guilt motivate support for reparation. Int. J. Conflict Viol. 4, 75–88. doi: 10.4119/UNIBI/IJCV.59

[ref2] Baumeister R. F. Stillwell A. M. Heatherton T. F. (1994). Guilt: an interpersonal approach. Psychol. Bull. 115, 243–267. doi: 10.1037/0033-2909.115.2.243, PMID: 8165271

[ref3] Behn R. D. (2001). Rethinking Democratic Accountability. Washington, DC: Brookings Institution Press.

[ref4] Berndsen M. Gausel N. (2015). When majority members exclude ethnic minorities: the impact of shame on the desire to object to immoral acts. Eur. J. Soc. Psychol. 45, 728–741. doi: 10.1002/ejsp.2127

[ref5] Branscombe N. R. (2004). “A social psychological process perspective on collective guilt,” in Collective Guilt: International Perspectives. eds. Branscombe R. Doosje B. (New York: Cambridge University Press), 320–334.

[ref6] Branscombe N. R. Doosje B. McGarty C. (2002). “Antecedents and consequences of collective guilt,” in From Prejudice to Intergroup Emotions: Differentiated Reactions to Social Groups. eds. Mackie D. M. Smith E. R. (New York, NY: Psychology Press), 49–66.

[ref7] Brown R. Čehajić S. (2008). Dealing with the past and facing the future: mediators of the effects of collective guilt and shame in Bosnia and Herzegovina. Eur. J. Soc. Psychol. 38, 669–684. doi: 10.1002/ejsp.466

[ref8] B’Tselem (2015). Unjustified Use of Lethal Force and Execution of Palestinians Who Stabbed or Were Suspected of Attempted Stabbings. Available at: https://www.btselem.org/gunfire/20151216_cases_of_unjustified_gunfire_and_executions (Accessed January 29, 2022).

[ref9] Buchman T. A. Tetlock P. E. Reed R. O. (1996). Accountability and auditors’ judgments about contingent events. J. Bus. Finan. Account. 23, 379–398. doi: 10.1111/j.1468-5957.1996.tb01128.x

[ref10] Burke J. C. (2005). Achieving Accountability in Higher Education: Balancing Public, Academic, and Market Demands. San Francisco, CA: Jossey-Bass.

[ref11] Carpenter C. Montgomery A. H. (2020). The stopping power of norms: saturation bombing, civilian immunity, and US attitudes toward the laws of war. Int. Security 45, 140–169. doi: 10.1162/isec_a_00392

[ref12] Čehajić S. Effron D. Halperin E. Liberman V. Ross L. (2011). Affirmation, acknowledgment of ingroup responsibility, group-based guilt, and support for reparative measures. J. Pers. Soc. Psychol. 101, 256–270. doi: 10.1037/a0023936, PMID: 21639648

[ref13] Chorev H. (2019). Palestinian social media and lone-wolf attacks: subculture, legitimization, and epidemic. Terror. Polit. Viol. 31, 1284–1306. doi: 10.1080/09546553.2017.1341878

[ref14] Condra L. N. Shapiro J. N. (2012). Who takes the blame? The strategic effects of collateral damage. Am. J. Polit. Sci. 56, 167–187. doi: 10.1111/j.1540-5907.2011.00542.x

[ref15] Crawford N. C. Lutz C. (2019). Human Cost of Post-9/11 Wars: Direct War Deaths in Major War Zones, Afghanistan and Pakistan (October 2001–October 2019) Iraq (March 2003–October 2019); Syria (September 2014–October 2019); Yemen (October 2002–October 2019); and Other. Watson Institute of International and Public Affairs at Brown University. Available at: https://watson.brown.edu/costsofwar/files/cow/imce/papers/2019/Direct/War/Deaths/COW/Estimate (Accessed November 13, 2019).

[ref16] Crozier W. (1998). Self-conscious in shame: the role of the “other”. J. Theory Soc. Behav. 28, 271–286. doi: 10.1111/1468-5914.00075

[ref17] Dearing R. L. Stuewig J. Tangney J. P. (2005). On the importance of distinguishing shame from guilt: relations to problematic alcohol and drug use. Addict. Behav. 30, 1392–1404. doi: 10.1016/j.addbeh.2005.02.002, PMID: 16022935 PMC3106346

[ref18] Deri A. R. (2012). “Costless” war: American and Pakistani reactions to the US drone war. Intersect 5, 1–16.

[ref19] Doosje B. E. Branscombe N. R. Spears R. Manstead A. S. (1998). Guilty by association: when one's group has a negative history. J. Pers. Soc. Psychol. 75, 872–886. doi: 10.1037/0022-3514.75.4.872

[ref20] Farooq T. Lucas S. Wolff S. (2020). Predators and peace: explaining the failure of the Pakistani conflict settlement process in 2013-4. Civil Wars 22, 26–63. doi: 10.1080/13698249.2020.1704603

[ref21] Friedrich J. Dood T. L. (2009). How many casualties are too many? Proportional reasoning in the valuation of military and civilian lives. J. Appl. Soc. Psychol. 39, 2541–2569. doi: 10.1111/j.1559-1816.2009.00537.x

[ref22] Fuhrman S. Elmore R. F. (eds.) (2004). Redesigning Accountability Systems for Education. Vol. 38. New York: Teachers College Press.

[ref23] Gallup (2011). Views of Violence. Available at: https://news.gallup.com/poll/157067/views-violence.aspx (Accessed January 29, 2022).

[ref24] Gausel N. Leach C. W. Mazziotta A. Feuchte F. (2018). Seeking revenge or seeking reconciliation? How concern for social-image and felt shame helps explain responses in reciprocal intergroup conflict. Eur. J. Soc. Psychol. 48, O62–O72. doi: 10.1002/ejsp.2295, PMID: 34420919

[ref25] Gausel N. Leach C. W. Vignoles V. L. Brown R. (2012). Defend or repair? Explaining responses to in-group moral failure by disentangling feelings of shame, rejection, and inferiority. J. Pers. Soc. Psychol. 102, 941–960. doi: 10.1037/a0027233, PMID: 22352324

[ref26] Gilbert P. Andrews B. (eds.) (1998). Shame: Interpersonal Behavior, Psychopathology, and Culture. Oxford, England: Oxford University Press. 78–98.

[ref27] Grant R. W. Keohane R. O. (2005). Accountability and abuses of power in world politics. Am. Polit. Sci. Rev. 99, 29–43. doi: 10.1017/S0003055405051476, PMID: 18469298

[ref28] Han S. Lerner J. S. Kattan M. (2009). “Accountability and medical decision making,” in The Encyclopedia of Medical Decision Making. Vol. 1. eds. Kattan M. W. Cowen M. E. (Thousand Oaks, CA: SAGE Publications).

[ref29] Harel A. Khouri J. Cohen G. (2016). Palestinian Killed in Stone-Throwing Incident Near Major Highway Was a Bystander, IDF Says. Haaretz. Available at: https://www.haaretz.com/israel-news/idf-palestinian-teen-killed-by-mistaken-gunfire-near-west-bank-highway-443-1.5399008 (Accessed January 29, 2022).

[ref30] Hautus M. J. (1995). Corrections for extreme proportions and their biasing effects on estimated values of d′. Behav. Res. Methods 27, 46–51. doi: 10.3758/BF03203619

[ref31] Hayes A. F. (2013). Introduction to Mediation, Moderation, and Conditional Process Analysis: A Regression-Based Approach. New York: The Guilford Press.

[ref32] Israel Ministry of Foreign Affairs (2018). Wave of Terror 2015–2018. Available at: http://mfa.gov.il/MFA/ForeignPolicy/Terrorism/Palestinian/Pages/Wave-of-terror-October-2015.aspx (Accessed January 29, 2022).

[ref33] Iyer A. Leach C. W. Crosby F. J. (2003). White guilt and racial compensation: the benefits and limits of self-focus. Personal. Soc. Psychol. Bull. 29, 117–129. doi: 10.1177/0146167202238377, PMID: 15272965

[ref34] Iyer A. Schmader T. Lickel B. (2007). Why individuals protest the perceived transgressions of their country: the role of anger, shame, and guilt. Personal. Soc. Psychol. Bull. 33, 572–587. doi: 10.1177/0146167206297402, PMID: 17400836

[ref35] Johns R. Davies G. A. (2019). Civilian casualties and public support for military action: experimental evidence. J. Confl. Resolut. 63, 251–281. doi: 10.1177/0022002717729733

[ref36] Jost J. T. Federico C. M. Napier J. L. (2009). Political ideology: its structure, functions, and elective affinities. Annu. Rev. Psychol. 60, 307–337. doi: 10.1146/annurev.psych.60.110707.163600, PMID: 19035826

[ref37] Kreps S. (2014). Flying under the radar: a study of public attitudes towards unmanned aerial vehicles. Res. Polit. 1:2053168014536533. doi: 10.1177/2053168014536533

[ref38] Krumpal I. (2013). Determinants of social desirability bias in sensitive surveys: a literature review. Qual. Quant. 47, 2025–2047. doi: 10.1007/s11135-011-9640-9

[ref39] Larson E. V. Savych B. (2007). Misfortunes of War: Press and Public Reactions to Civilian Deaths in Wartime. Santa Monica, CA: Rand Corporation.

[ref40] Leach C. W. Cidam A. (2015). When is shame linked to constructive approach orientation? A meta-analysis. J. Pers. Soc. Psychol. 109, 983–1002. doi: 10.1037/pspa0000037, PMID: 26641074

[ref41] Leith K. P. Baumeister R. F. (1998). Empathy, shame, guilt, and narratives of interpersonal conflicts: guilt-prone people are better at perspective taking. J. Pers. 66, 1–37. doi: 10.1111/1467-6494.00001

[ref42] Lerner J. S. Goldberg J. H. Tetlock P. E. (1998). Sober second thought: the effects of accountability, anger, and authoritarianism on attributions of responsibility. Personal. Soc. Psychol. Bull. 24, 563–574. doi: 10.1177/0146167298246001

[ref43] Lerner J. S. Shonk K. (2006). Create accountability, improve negotiations. Negotiation 9, 1–4.

[ref44] Lerner J. S. Tetlock P. E. (1999). Accounting for the effects of accountability. Psychol. Bull. 125, 255–275. doi: 10.1037/0033-2909.125.2.255, PMID: 10087938

[ref45] Lewis H. B. (1971). Shame and Guilt in Neurosis. New York: International Universities Press.

[ref46] Lickel B. Schmader T. Barquissau M. (2004). “The evocation of moral emotions in intergroup contexts: the distinction between collective guilt and collective shame,” in Collective Guilt: International Perspectives. eds. Branscombe N. Doojse B. (Cambridge: Cambridge University Press), 35–55.

[ref47] Lickel B. Steele R. R. Schmader T. (2011). Group-based shame and guilt: emerging directions in research. Soc. Personal. Psychol. Compass 5, 153–163. doi: 10.1111/j.1751-9004.2010.00340.x

[ref48] Lyall J. Blair G. Imai K. (2013). Explaining support for combatants during wartime: a survey experiment in Afghanistan. Am. Polit. Sci. Rev. 107, 679–705. doi: 10.1017/S0003055413000403

[ref49] Mashuri A. van Leeuwen E. (2020). Promoting reconciliation in separatist conflict: the effect of morality framing. Group Process. Intergr. Relat. 24, 1200–1218. doi: 10.1177/1368430220934856

[ref50] Miller S. D. (1995). Teachers’ responses to test-driven accountability pressures: if i change, will my scores drop? Read. Res. Instr. 34, 332–351. doi: 10.1080/19388079509558190

[ref51] Mosquera P. M. R. Manstead A. S. Fischer A. H. (2002). The role of honour concerns in emotional reactions to offences. Cognit. Emot. 16, 143–163. doi: 10.1080/02699930143000167

[ref52] Pew Research Center (2015). Public Continues to Back U.S. Drone Attacks. Available at: http://www.people-press.org/2015/05/28/public-continues-to-back-u-s-drone-attacks/ (Accessed January 29, 2022).

[ref53] Pew Research Center (2017). Few in U.S. Say it Can be Justifiable to Target and Kill Civilians. Available at: http://www.pewforum.org/2017/07/26/terrorism-and-concerns-about-extremism/pf_2017-06-26_muslimamericans-05-01/ (Accessed January 29, 2022).

[ref54] Przeworski A. Stokes S. C. Manin B. (eds.) (1999). Democracy, Accountability, and Representation (Vol. 2). Cambridge: Cambridge University Press.

[ref55] Rees J. H. Allpress J. A. Brown R. (2013). Nie wieder: group-based emotions for in-group wrongdoing affect attitudes toward unrelated minorities. Polit. Psychol. 34, 387–407. doi: 10.1111/pops.12003

[ref56] Retzinger S. M. (1987). “Resentment and laughter: video studies of the shame-rage spiral,” in The Role of Shame in Symptom Formation. ed. Lewis H. B. (Hillsdale, NJ: Lawrence Erlbaum Associates), 151–181.

[ref57] Ryan M. (2018). Civilian Deaths Tripled in U.S.-Led Campaign Against ISIS in 2017, Watchdog Alleges. Washington Post. Available at: https://www.washingtonpost.com/world/national-security/civilian-deaths-tripled-in-us-led-campaign-during-2017-watchdog-alleges/2018/01/18/ccfae298-fc6d-11e7-a46b-a3614530bd87_story.html?noredirect=on&utm_term=.84c686add1ec (Accessed January 29, 2022).

[ref58] Sagan S. D. Valentino B. A. (2017). Revisiting Hiroshima in Iran: what Americans really think about using nuclear weapons and killing noncombatants. Int. Security 42, 41–79. doi: 10.1162/ISEC_a_00284

[ref59] Schori-Eyal N. Halperin E. Saguy T. (2019). Intergroup commonality, political ideology, and tolerance of enemy collateral casualties in intergroup conflicts. J. Peace Res. 56, 425–439. doi: 10.1177/0022343318818658

[ref60] Scott M. B. Lyman S. (1968). Accounts. Am. Sociol. Rev. 33, 46–62. doi: 10.2307/2092239, PMID: 5644339

[ref61] Semin G. R. Manstead A. S. R. (1983). The Accountability of Conduct: A Social Psychological Analysis. New York: Academic Press.

[ref62] Shah A. (2018). Do US drone strikes cause blowback? Evidence from Pakistan and beyond. Int. Security 42, 47–84. doi: 10.1162/isec_a_00312

[ref63] Shalev A. Y. Peri T. Rogel-Fuchs Y. Ursano R. J. Marlowe D. (1998). Historical group debriefing after combat exposure. Mil. Med. 163, 494–498. doi: 10.1093/milmed/163.7.494, PMID: 9695618

[ref64] Shaver A. Shapiro J. (2015). The effect of civilian casualties on wartime informing: Evidence from the Iraq war. J. Confl. Resolut. Forthcoming.

[ref65] Shepherd L. Spears R. Manstead A. S. (2013a). The self-regulatory role of anticipated group-based shame and guilt in inhibiting in-group favoritism. Eur. J. Soc. Psychol. 43, 493–504. doi: 10.1002/ejsp.1971

[ref66] Shepherd L. Spears R. Manstead A. S. (2013b). When does anticipating group-based shame lead to lower ingroup favoritism? The role of status and status stability. J. Exp. Soc. Psychol. 49, 334–343. doi: 10.1016/j.jesp.2012.10.012

[ref67] Shepherd L. Spears R. Manstead A. S. (2013c). ‘This will bring shame on our nation’: the role of anticipated group-based emotions on collective action. J. Exp. Soc. Psychol. 49, 42–57. doi: 10.1016/j.jesp.2012.07.011 23690650 PMC3657186

[ref68] Sibley C. G. Duckitt J. (2008). Personality and prejudice: a meta-analysis and theoretical review. Personal. Soc. Psychol. Rev. 12, 248–279. doi: 10.1177/1088868308319226, PMID: 18641385

[ref69] Skitka L. J. McMurray P. J. Burroughs T. E. (1991). Willingness to provide post-war aid to Iraq and Kuwait: an application of the contingency model of distributive justice. Contemp. Soc. Psychol. 15, 179–188.

[ref70] Slovic P. Mertz C. K. Markowitz D. M. Quist A. Västfjäll D. (2020). Virtuous violence from the war room to death row. Proc. Natl. Acad. Sci. U. S. A. 117, 20474–20482. doi: 10.1073/pnas.2001583117, PMID: 32778580 PMC7456115

[ref71] Smith R. H. Webster J. M. Parrott W. G. Eyre H. L. (2002). The role of public exposure in moral and nonmoral shame and guilt. J. Pers. Soc. Psychol. 83, 138–159. doi: 10.1037/0022-3514.83.1.138, PMID: 12088123

[ref72] Stanislaw H. Todorov N. (1999). Calculation of signal detection theory measures. Behav. Res. Methods Instrum. Comput. 31, 137–149. doi: 10.3758/BF03207704, PMID: 10495845

[ref73] Stenning P. C. (ed.) (1995). Accountability for Criminal Justice. Toronto, Canada: University of Toronto Press.

[ref74] Stuewig J. Tangney J. P. Heigel C. Harty L. McCloskey L. (2010). Shaming, blaming, and maiming: functional links among the moral emotions, externalization of blame, and aggression. J. Res. Pers. 44, 91–102. doi: 10.1016/j.jrp.2009.12.005, PMID: 20369025 PMC2848360

[ref75] Tangney J. P. Dearing R. L. (2002). Shame and Guilt. New York: Guilford Press.

[ref76] Tangney J. P. Stuewig J. Mashek D. J. (2007). Moral emotions and moral behavior. Annu. Rev. Psychol. 58, 345–372. doi: 10.1146/annurev.psych.56.091103.070145, PMID: 16953797 PMC3083636

[ref77] Tetlock P. E. (1983). Accountability and complexity of thought. J. Pers. Soc. Psychol. 45, 74–83. doi: 10.1037/0022-3514.45.1.74, PMID: 34336611

[ref78] Tetlock P. E. (1992). The impact of accountability on judgment and choice: toward a social contingency model. Adv. Exp. Soc. Psychol. 25, 331–376. doi: 10.1016/s0065-2601(08)60287-7

[ref79] Tracy J. L. Robins R. W. (2004). Putting the self into self-conscious emotions: a theoretical model. Psychol. Inq. 15, 103–125. doi: 10.1207/s15327965pli1502_01

[ref81] Uhlmann E. L. Pizarro D. A. Tannenbaum D. Ditto P. H. (2009). The motivated use of moral principles. Judgm. Decis. Mak. 4, 479–491.

[ref82] US Army (2011). Leader’s Guide to After-Action Reviews (AAR). US Army Combined Arms Center-Training: Fort Leavenworth, Kansas.

[ref80] U.S. Joint Chiefs of Staff (2013). Joint Targeting (Joint Publication 3–60). Washington, DC: U.S. Joint Chiefs of Staff. Available at: https://www.justsecurity.org/wp-content/uploads/2015/06/Joint_Chiefs-Joint_Targeting_20130131.pdf (Accessed January 29, 2022).

[ref83] Vashdi D. R. Bamberger P. A. Erez M. Weiss-Meilik A. (2007). Briefing-debriefing: using a reflexive organizational learning model from the military to enhance the performance of surgical teams. Hum. Resour. Manag. 46, 115–142. doi: 10.1002/hrm.20148

[ref84] Walsh J. I. (2015). Precision weapons, civilian casualties, and support for the use of force. Polit. Psychol. 36, 507–523. doi: 10.1111/pops.12175

[ref85] Wayne C. Porat R. Tamir M. Halperin E. (2016). Rationalizing conflict: the polarizing role of accountability in ideological decision making. J. Confl. Resolut. 60, 1473–1502. doi: 10.1177/0022002714564431

