# Supplementary material for: Shamed If You Do, Shamed If You Do Not: Group-Based Moral Emotions, Accountability, and Tolerance of Enemy Collateral Casualties
Source: Front Psychol. 2022 Mar 2;13:750548. doi: 10.3389/fpsyg.2022.750548 (PMC8924286; doi:10.3389/fpsyg.2022.750548)
Supplement: Supplementary file 1 [file Table_1.DOCX]

**Supplementary Material**

**Forecast group-based moral emotions: Study 1**

*When members of security forces or civilians try to stop a terror attack as it unfolds, uninvolved civilians are sometimes harmed. If during a terror event an uninvolved civilian (i.e., a civilian who is in no way associated with the terror event) is hurt by actions aimed at stopping the terrorist, to what extent do you think you would experience each of the following emotions?*

1. Anger at the Palestinians
2. Pride over the attempt to stop the attack
3. Hatred towards the Palestinians
4. Guilt over the harm caused to an uninvolved civilian
5. Pity towards the Palestinians
6. Joy over the attempt to stop the attack
7. Empathy towards the Palestinians
8. Shame over the harm caused to an uninvolved civilian
9. Fear of the Palestinians
10. Pity towards the uninvolved civilian harmed in the event

* Forecast emotions were presented at a random order

**Forecast group-based moral emotions: Study 2**

*When members of security forces or civilians try to stop a terror attack as it unfolds, uninvolved civilians are sometimes harmed. If during a terror event an uninvolved civilian (i.e., a civilian who is in no way associated with the terror event) is hurt by actions aimed at stopping the terrorist, to what extent do you think you would experience each of the following emotions?*

I forecast I would feel **shame** over harm to Palestinian civilians during the action

I forecast I would feel **anger** at the Palestinians

I forecast I would feel **guilt** over harm to Palestinian civilians during the action

I forecast I would **pride** over the Israeli response to the attack.

I forecast I would feel **fear** of the Palestinians.

I forecast I would feel **hatred** towards the Palestinians.

I forecast I would feel **empathy** towards the Palestinian civilians harmed during the action

I forecast I would feel **remorse** over harm to Palestinian civilians during the action

* Forecast emotions were presented at a random order

**Forecast group-based moral emotions: Study 2**

*As an IDF soldier or officer you may often encounter events in which you, independently or as part of your unit, are required to use force on Palestinians (directly or indirectly) in order to ensure the security of Israeli citizens. In such events, and as part of the attempt to stop terror attacks, Palestinian citizens who are not involved in fighting can sometimes be harmed. If during an attempt to stop a terror attack or during a military action in which you took part uninvolved civilians were harmed, how much do you forecast you would feel each of the following emotions?*

I forecast I would feel **shame** over harm to Palestinian civilians during the action

I forecast I would feel **anger** at the Palestinians

I forecast I would feel **guilt** over harm to Palestinian civilians during the action

I forecast I would **pride** over the Israeli response to the attack.

I forecast I would feel **fear** of the Palestinians.

I forecast I would feel **hatred** towards the Palestinians.

I forecast I would feel **empathy** towards the Palestinian civilians harmed during the action

I forecast I would feel **remorse** over harm to Palestinian civilians during the action

**Accountability manipulation (Study 1)**

*Accountability condition:*

In this study we are interested in how people respond to the current wave of terrorism. First we will ask you to read descriptions of events that recently took place and respond to them – answer a few questions about your thoughts, attitudes and emotions regarding these scenarios, as well as the suitable course of action. After you complete the questionnaire you will be interview by Dr. Ephraim Cohen, a senior researcher at Tel-Aviv University who has been working for many years on the topic of decision making in conflicts, and is collaborating with us on this research. During the interview, Dr. Cohen will ask you to describe in detail the rationale behind your decisions in the scenarios presented.

*Control condition:*

In this study we are interested in how people respond to the current wave of terrorism. First we will ask you to read descriptions of events that recently took place and respond to them – answer a few questions about your thoughts, attitudes and emotions regarding these scenarios, as well as the suitable course of action. It's important for me to emphasize that we ask you to write down your user name so that we can match your responses to the preliminary survey you completed, but it will be separated from your answers. Anonymity will be complete and we will have no way of knowing who wrote what.

**Accountability manipulation (Study 2)**

**Accountability prime**

One of the unique characteristics of the army as an organization is the issue of debriefings and the emphasis on learning from military operations. In this part of the study, we ask you think of all the contexts in which you, as part of your military service, undergo debriefings and are required to explain your actions to your commanders or to other military personnel. Please describe two recent events in which you were debriefed and explained in detail (in writing or face-to-face) why you acted the way you did, as a soldier or an officer. Please write whether the debriefing was conducted at the individual or team level; who was present at the debriefing and who managed it; to whom were the conclusions reported. There is no need to write specific names or positions – you may use descriptions like "the commander of the unit" or "soldiers in the team".

**Control**

In this part of the study, we ask you to think of all the contexts in which you, as part of your military service, participate in physical exercise and learn new skills related to sport and physical activity. Please describe two recent occasions in which you exercised in the army, individually or as part of a group. Please write what was the physical activity, who participated it, where it was held, and what was the main skill practiced.

**Tolerance of enemy collateral casualties: Study 1**

The scenarios you will now read describe events similar to recent terror attacks that took place in Israel. For each scenario, we ask that you try to imagine that you are on the scene, and what your response would be. Remember! We are interested in the answer that most honestly and accurately reflects your feelings and the way you think you would respond to the event. *

- You are walking down a crowded street. You see a man ahead of you collapse. He is bleeding from multiple stab wounds. Next to him you see a man wearing a keffiyeh holding a bloody knife. **
- You are waiting for a train. Suddenly you realize that further down the platform, someone has been stabbed, and has collapsed onto the ground. Not far from where they are laying you can see a woman in a hijab, who is holding something in her hand, but from where you are standing you can’t tell exactly what.
- You hear cries and screams in a crowded bus station, and run to see what has happened. When you arrive people are crowded around an injured man trying to stop the bleeding. As you arrive, you see a darker-skinned man backing away into the crowd.
- You are walking to the market, and suddenly hear a loud crash. You turn the corner and see that a car has crashed into a bus stop. You can’t tell if anyone has been hurt but you can see a man getting out of the car that crashed.
- You are walking home from work a night and see an Arab man running towards you, being chased by a group of men in yarmulkes [Jewish religious headwear] who are yelling, “Terrorist! Terrorist!!”
- On your way back from visiting an acquaintance in Gush Etzion [in the West Bank], you are passing through a checkpoint where Palestinians are also passing. From your car you see a Palestinian woman approaching a soldier. As she approaches she reaches into her purse and pulls something out. Then another car blocks your view.
- While driving to the Dead Sea for a vacation day, you arrive at a checkpoint. While you are waiting to pass an older Palestinian woman crashes her car into the roadblock in front of the checkpoint.
- You are shopping in the market and here screams, you rush towards them and when you arrive you see victim who has been stabbed multiple times and an Arab man bending over him trying to stop the bleeding with his hands calling for help.

* scenarios were presented at random order

** this scenario was coded as a "hit" (signal trial)

**Tolerance of enemy collateral casualties: Study 2**

In the next part of the study we would like to examine aspects of operational decision making during the military service. In Israel's current state of security, many people – mostly members of the security forces – face dilemmas and have to make difficult decisions that affect the lives of Israelis and Palestinians alike. We will now present you with descriptions of several events based on real events that took place and recent months, and ask you to indicate which decision you would make in each case if you experienced it yourself during your military service.

- You are walking to the market, and suddenly hear a loud crash. You turn the corner and see that a car has crashed into a bus stop. It looks as though at least three people have fallen down injured, and you can see a man getting out of the car that crashed.
- You are returning on foot from a night shift at the military base and suddenly see an Arab man running towards you, being chased by a group of men in yarmulkes [Jewish religious headwear] who are yelling, “Terrorist! Terrorist!!”
- You are standing at a crowded bus station, when suddenly you hear screams and cries of distress right behind you. You turn around and see several people lying on the ground, bleeding from stab wounds, and a young man who looks very stressed, backing away and trying to lose himself in the crowd.
- You are waiting for a bus on your way to the military base, when suddenly you hear the crash of metal and turn to see that someone rammed into a bus station on the other side of the road. Several people are injured, and the car is moving in reverse as though the driver wants to escape or to run over the injured people at high speed.
- You are shopping in the market when suddenly you hear a gunshot. You run towards the source of the noise and see a young man standing over a body. He is holding a gun, trembling, he seems very agitated and looks all around.
- You are waiting for a train. Suddenly you realize that further down the platform, someone has been stabbed, and has collapsed onto the ground. Not far from where they are laying you can see a woman in a hijab, who is holding a sharp-looking object, but from where you are standing you can’t tell whether it's a knife, a screwdriver, or something else.
- You serve in a checkpoint near Jerusalem. You see a young Palestinian woman who is walking down a vehicle lane (rather than a pedestrian lane). You call to her and instruct her to stop, but she continues to walk quickly and tries to extract something from her handbag.
- You are manning a checkpoint in the West Bank, when suddenly a Palestinian woman in her seventies crashes into the roadblock in front of the checkpoint.

**Calculation of TECC measure**

The loglinear correction is common approach for correcting for extreme values in signal detection measurements, it involves adding .5 to the number of hits and false alarms and 1 to the number of signal trials and 1 to the number of noise trials. It is needed because if the hit or false alarm rate is 0 or 1, mathematically the bias score cannot be determined because 0 and 1 correspond to z scores of negative infinity or infinity. Because shooting with the intent to kill is an extreme and serious action there were many participants who did not choose this option for any scenario, resulting in a hit rate of 0. Therefore some correction for extreme values was necessary. We chose the loglinear approach because it is a common and effective way of dealing with extreme values (Hautus, 1995) and it can be applied even in the absence of extreme values (Stanislaw & Todorov, 1999). Therefore, we applied it to all bias scores.

**Excluded Study: Failed Manipulation**

**Participants and Procedure**

One hundred and ten Jewish-Israeli respondents were recruited through an online survey panel and completed the study in exchange for a small monetary compensation^[[1]](#footnote-1)^^[[2]](#footnote-2)^. The sample included 62 men and 48 women whose ages ranged between 18 and 74, *M*=42.21, *SD*=15.90. After completing a measure of forecast group-based emotions, participants were randomly assigned to read one of two texts: either about the rising prevalence and importance of debriefings in organizations (accountability condition) or about millennials in the workplace (control condition). The prime was composed to bring to participants’ awareness central features of accountability and its benefits, as reflected in the process of organizational debriefing procedures. After reading the text and answering comprehension questions, participants completed the same set of vignettes described in Study 1. Finally, they answered demographic questions and were debriefed about the study.

**Measures**

The **forecast group-based emotions** measure was similar to the one described in Study 1, except that the list of emotions was shorter (8 emotions, including shame and guilt^[[3]](#footnote-3)^). The **accountability manipulation** was described as a study that examined how people process information provided in different media. The accountability text, supposedly an article taken from a financial newspaper website, described the rising prevalence of formal debriefing procedures in various organizations, and delineated their benefits as described by an organizational psychologist interviewed in the article (see SM for full text). The control condition was supposedly from the same website but described the challenges and benefits of integrating millennial employees in the workforce. **Tolerance of enemy collateral casualties** **(TECC)** and **political orientation** were identical to those described in the previous study.

**Results and Discussion**

We again used PROCESS (Hayes, 2013) to examine the effects of forecast shame and accountability on TECC^[[4]](#footnote-4)^. Results of the analysis with forecast shame as a moderator indicated that forecast shame (*b*=.04, *SE*=.03, *t*=1.15, *p*=.14) and experimental condition (*b*=.09, *SE*=.07, *t*=1.37, *p*=.17) were not significant predictors of TECC. After considering the main effects of forecast shame and experimental condition, we found that as in the previous study the interaction between them was a significant predictor of TECC (*b*=-.11, *SE*=.05, *t*=-2.00, *p*=.049; see Figure 2). Analysis of the simple effects indicated that again participants with high levels of forecast shame were unaffected by the experimental condition (*b*=-.08, *SE*=.11, *t*=-.76, *p*=.45). In contrast, participants with low levels of forecast shame who were primed with accountability by reading about the importance of accountability were less likely to make the “shoot” decision compared with those in the control condition (*b* =.23, *SE*=.1, *t*=2.38, *p*=.02). The difference between low- and high- forecast shame was significant in the control condition (*b*=.1, *SE*=.04, *t*=2.46, *p*=.02), but not significant in the accountability condition (*b*=-.01, *SE*=.04, *t*=-.12, *p*=.90). The entire model was marginally significant (*r^2^*^=^.07, *F* (4, 104)=2.04, *p*=.095). We then repeated the analysis with forecast guilt as the moderator. Neither the experimental condition (*b* =.1, *SE*=.07, *t*=1.37, *p*=.17) nor of forecast guilt (*b* =.01, *SE*=.03, *t*=.35, *p*=.73) nor the interaction between them (*b* =-.08, *SE*=.06, *t*=1.48, *p*=.15) were significant predictors of TECC, and the entire model was not significant (*r^2^*^=^.04, *F* (4, 104)=1.07, *p*=.38).

**Accountability manipulation (Failed)**

**Accountability Prime**

***How to turn organizational failures into successes: Debriefings are the key***

A dominant trend in the business world in recent years is the significant rise in internal debriefings in the private and public sector. A debriefing is defined as a systematic process for understanding circumstances and details of an event (what happened, how did and happen and why did it happened) while extracting the most data and information, with the goal of creating reliable infrastructure for reaching conclusions and forming lessons. After debriefing those involved and writing an event report, recommendations are given to the executive tier to decide what to implement and in what way. Debriefings are an inseparable part of activity in systems like the health system, in which debriefings are carried out after medical procedures with the goal of training medical teams and improving their skills; and the security system, in which operational debriefings of soldiers and officers by their commanders is an essential part of the operational routine.

In the last two decades, internal debriefings after events such as a widespread technical malfunction or a significant service failure have spread, and many companies now incorporate debriefings into the organizational culture. Dr. Ethan Perry, an organizational psychologist and business consultant, explains: “a high-quality debriefing can improve financial efficiency, function, work environment and customer service while increasing the client-organization trust. A debriefing report can also reflect what’s going on in the organization to high-level managers and underline difficulties that harm the organization’s ability to function”.

**What constitutes a high-quality debriefing?**

“several principles make debriefing a positive process within the organization, rather than a scary ‘witch hunt’. A debriefing should be conducted as close as possible to the event and requires cooperation and dedication of the entire organization, in order to examine processes and not look for someone to blame. Good debriefing necessitates full knowledge of the event, in order to identify weak links in the decision-making process and to prevent future failures. It’s important to debrief in a fair, objective, professional manner, while maintaining open discourse and out of the desire to improve the work – not a desire to find malfunctions and guilty workers at all costs, but with openness and transparency. When an organization is able to incorporate a healthy environment of debriefings, we see that employees and managers take greater responsibility over their actions and think more deeply while making decisions – and that’s something any company can benefit from.

**What would you suggest to a manager who wants to start debriefing in her company?**

“first of all, explain the goal of the debriefing to the employees. You have to be very clear that the goal is not to find workers at fault but to improve their capabilities and enable the organization to operate more efficiently. I also suggest to implement debriefings in a gradual, non-threatening manner, and to be alert to employees’ and managers’ feelings about it, because it’s a significant change”.

**Control:**

***How to bridge generation gaps in the organization: Direct and honest communication is the key***

A dominant trend in the business world in recent years is the presence of “Millennials”, or Y-generation, and effect on the workplace in the public and private sector. Millennials are loosely defined as those born between 1982-2004, and as their percentage in the workplace increases so does their impact on the companies and organizations in which they work. On the one hand, these young employees tend to be less committed to their workplace, have shorter attentions spans and gaps in general knowledge compared with older employees, on the other hand, their dynamism in the job market and their ease with technology and globalization are one the important factors behind many changes we’ve witnessed in the last decade.

Are there principles or guidelines that can assist managers in incorporating Millennials in the workplace? Dr. Ethan Perry, an organizational psychologist and business consultant, explains: “these young employees can pose a challenge to older managers. They can be confused, authentic to the point of impertinence, sometimes disoriented to their surroundings. But they are young and energetic, willing to challenge the institution, and think openly and creatively. You have to know how to manage them in a way that would make the most of their advantages and strength, and there is no doubt that any company can benefit from incorporating your employees in the workforce.

**How to deal with such a generational gap?**

“Several principles help managing Millennials and avoiding miscommunication and the creation of opposing camps, “older generation” vs. “younger generation”. It’s important to communicate authentically, clearly and without manipulations. They’re interested in ongoing feedback and open dialogue with their managers, and they pull back if they receive only criticism over the way they function. It’s very important to give them a sense of meaning and to create enthusiasm about their position, because young employees are not afraid to move from one company to another in order to get what’s important to them: financial benefits as well as a sense of meaning.

**And what are the advantages or unique outputs of “Millennial” employees?**

“There is much to learn from this generation: flexibility, diversity, creativity, and thinking outside the box. Young employees grew up in a less hierarchical world, a flat world in which everyone contributes equally to the creation of knowledge. Their openness to technological changes, their mental flexibility and their willingness to embrace difference and cultural diversity are characteristics that can benefit any company that employs them, if their managers knows how to harness them effectively”.

1. The original sample size was N=200; 90 participants who did not complete the manipulation according to instructions or failed the attention checks were excluded from analyses. [↑](#footnote-ref-1)
2. Assuming a two-tailed alpha of .05 and standard power (80%) we had power to detect on effect of change in r-squared of .067. [↑](#footnote-ref-2)
3. Additional forecast group-based emotions included anger, pride, hatred, fear, empathy and remorse. As in Study 1, none of the emotions predicted TECC or moderated the effect of accountability on TECC. [↑](#footnote-ref-3)
4. As in Study 1, we examined all variables for outliers, and excluded one participant who was an extreme outlier (SD > 2.5) on the measure of TECC. [↑](#footnote-ref-4)
